# Model of vaccine efficacy against HSV-2 superinfection of HSV-1 seropositive mice demonstrates protection by antibodies mediating cellular cytotoxicity

**DOI:** 10.1038/s41541-020-0184-7

**Published:** 2020-05-07

**Authors:** Clare Burn Aschner, David M. Knipe, Betsy C. Herold

**Affiliations:** 1 Department of Microbiology and Immunology, Albert Einstein College of Medicine, Bronx, NY 10461 USA; 2 Department of Microbiology, Blavatnik Institute, Harvard Medical School, Boston, MA 02115 USA; 3 Department of Pediatrics, Albert Einstein College of Medicine, Bronx, NY 10461 USA

**Keywords:** Infection, Infectious diseases, Vaccines, Vaccines, Virology

## Abstract

A majority of the world’s population is infected with HSV-1, highlighting the need for vaccines that are effective in HSV-1-seropositive hosts. We established a superinfection model by infecting mice intranasally with a sublethal dose of HSV-1, which results in high rates of seropositive, latently infected mice susceptible to HSV-2 superinfection. Sublethal HSV-1 induced a predominantly neutralizing antibody response. Vaccination of HSV-1-seropositive mice with recombinant adjuvanted glycoprotein D (rgD-2) failed to significantly boost HSV total or neutralizing antibody responses and provided no significant increased protection against HSV-2 superinfection compared to control-vaccinated HSV-1-seropositive mice. In contrast, immunization with a single-cycle virus deleted in gD (ΔgD-2) significantly boosted total HSV-specific antibody titers and elicited new antibody-dependent cell-mediated cytotoxicity responses, providing complete protection from death following HSV-2 superinfection. This model recapitulates clinical responses to natural infection and the rgD-2 vaccine trial outcomes and suggests that ΔgD-2 may prove protective in HSV-1-seropositive hosts.

## Introduction

Herpes simplex virus 1 and 2 (HSV-1 and HSV-2) are prevalent infections causing recurrent oral, ocular and genital disease as well as sporadic encephalitis^1,2^. HSV-1 is the predominant cause of oral and ocular disease, while both serotypes cause genital disease^1^. In the U.S, HSV-1 has emerged as the more common cause of genital herpes, but in Africa, HSV-2 continues to dominate and numerous epidemiologic studies have shown that genital HSV-2 infection contributes more than any other biological factor to the HIV epidemic^2–7^. Mathematical modeling suggests that a vaccine that prevents HSV-2 would also have a major impact on HIV acquisition and transmission^4,7^. Worldwide, it is estimated that 67% of the population is infected with HSV-1 by 49 years of age, with many in developing countries being infected early in childhood^6^. Thus, a vaccine for HSV-2 prevention must be immunogenic and protective in HSV-1 seropositive individuals.

Several prophylactic HSV-vaccine candidates have been evaluated in clinical trials with overall disappointing results. A subunit vaccine comprised of recombinant HSV-2 glycoprotein D combined with alum (aluminum and magnesium hydroxide) and monophosphoryl lipid A (MPL) (gD-AS04) was evaluated in three different Phase 3 clinical trials. The first two studies were conducted in serodiscordant couples and included HSV-1-seropositive individuals in the second consort^8^. The vaccine showed no efficacy in HSV-1-seropositive men or women, but provided some protection against HSV-2 acquisition in doubly (HSV-1 and HSV-2)-seronegative women, but not men^8^. However, in a subsequent field study in which only doubly-seronegative women were enrolled, there was no protection against genital HSV-2 but modest protection against genital HSV-1^9^.

Preclinical studies conducted in HSV-1-seropositive guinea pigs had demonstrated that an adjuvanted recombinant gD-2 (rgD-2) vaccine (two doses each containing 3 µg of rgD-2 combined with 75 µg alum and 7.5 µg MPL) boosted neutralizing antibody responses and protected guinea pigs from subsequent intravaginal challenge with HSV-2^10^. These studies, which did not predict the Phase 3 clinical trial outcome, were conducted using laboratory-adapted isolates of HSV-1 (strain KOS) and HSV-2 (strain 333). A model testing vaccine efficacy against HSV-2 superinfection of HSV-1-seropositive mice has not been previously published, but it has been shown that mice are susceptible to heterologous HSV challenge at sites distant from the site of primary infection^11^.

We recently engineered an HSV-2 virus deleted in gD, and found that this single-cycle viral candidate vaccine is safe and provides potent protection in female and male naïve mice challenged with lethal doses of clinical isolates of HSV-1 and HSV-2^12–14^. In contrast, an adjuvanted rgD-2 subunit vaccine (2 doses each containing 5 µg of rgD-2 combined with 150 µg alum and 12.5 µg MPL herein referred to as rgD-2/alum-MPL) provided only modest protection when the mice were challenged with these clinical isolates^14^. The subunit vaccine induced a predominantly neutralizing antibody response in naïve (seronegative) mice, whereas ΔgD-2 induced a predominantly non-neutralizing, Fc gamma receptor (FcγR)-activating antibody response that mediated antibody-dependent cell-mediated cytotoxicity (ADCC) and phagocytosis^12–14^.

Building on these observations, we established and characterized a model of HSV-2 superinfection of HSV-1-seropositive mice using clinical isolates, and then compared the immunogenicity and efficacy of ΔgD-2 and rgD-2/alum-MPL in this new model. We further tested the model using HSV-2 *dl5-29*, a replication defective candidate viral vaccine strain, which recently completed a Phase 1 clinical trial^15^. In HSV-1-seropositive guinea pigs, *dl5-29* significantly boosted the neutralizing antibody titer and provided protection against HSV-2 superinfection, although the responses were similar to what was observed with adjuvanted rgD-2 vaccine. ADCC antibodies were not measured. In contrast, in the Phase 1 *dl5-29* trial, vaccination of HSV-1-seropositive individuals resulted in an initial increase in neutralizing titers, but the response was not sustained and there was no significant difference compared to baseline neutralizing titers 30 days after the third vaccine dose^15^.

## Results

### Establishment of a model of HSV-2 superinfection of HSV-1-seropositive mice

**Fig. 1:**
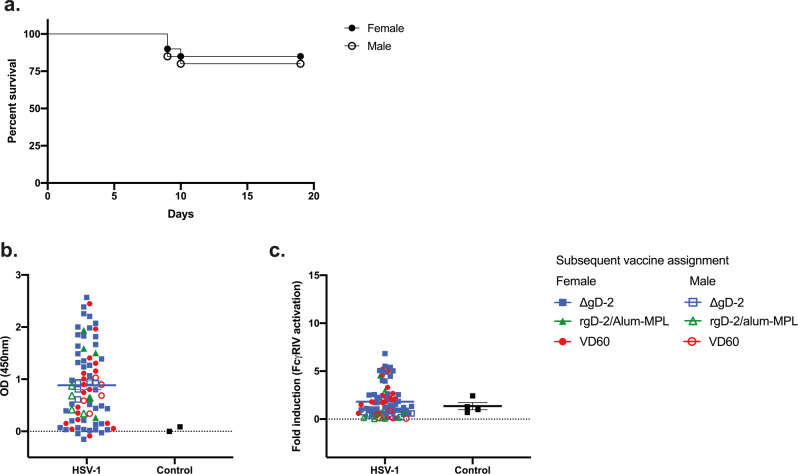
Establishment and characterization of a mouse model of HSV-1 seropositivity. **a** Female (closed symbols) and male (open symbols) C57BL6 mice were infected intranasally with 5 × 10^4^ pfu/mouse of HSV-1 B^3^ × 1.1. Mice were monitored over 20 days for signs of disease and distress. At day 14 post-primary challenge, mice were bled retro-orbitally to obtain immune serum, which was tested for **b** total HSV-1-specific IgG by ELISA (1:10,000 dilution) and **c** fold-induction of FcγRIV activation (mFcγRIV ADCC Reporter Bioassay, Promega). Data points are shown with symbols to indicate vaccination groups to which each mouse was subsequently assigned; the mean is indicated. Panel **a**, *n* = 75 females and 20 males; Panel **b**, *n* = 67 females and 16 males across four independent experiments.

Intranasal inoculation of mice with 5 × 10^4^ pfu of the clinical isolate, HSV-1 B^3^ × 1.1, consistently resulted in 80–90% survival in female (closed symbols) and male (open symbols) mice with high rates of seroconversion (Figs. 1a, b). Among 75 female mice, 67 survived and 64/67 seroconverted and had detectable HSV-1-specific Abs in their serum by ELISA. Similarly, 16/20 male mice survived and 15/16 seroconverted. The HSV-1 IgG endpoint dilution titers were variable and did not differ comparing males and females (Table 1 and Fig. 1b).

**Table 1:** Antibody responses to primary HSV-1 infection.

Assay	Median	Range	*n*
HSV-1 IgG			
Female	30,000	1000–270,000	63
Male	90,000	10,000–270,000	15
Neutralizing Titer			
Female	200	25–400	18
Male	200	50–400	10
FcγRIV activation			
Female	1.345	0.01–6.83	63
Male	0.735	0.05–5.39	15
gB-1 IgG (Female)	270,000	10,000–270,000	16
gD-1 IgG (Female)	27, 000	30,000–270,000	32

HSV-1, gB-1, and gD-1 IgG titers were determined by ELISA and are reported as endpoint dilutions of immune serum (cutoff optical density units at 450 nm of 0.2). Neutralizing titers are defined as the highest dilution of immune serum that inhibited 50% of HSV-1 viral plaque formation. The FcγRIV activation is reported as fold-induction after subtracting background.

To evaluate the functionality of Abs elicited in response to sublethal HSV-1 infection, the neutralizing antibody titer (Table 1) and ability to activate FcγRIV as a biomarker of ADCC (Fig. 1c) were determined using HSV-1 B^3^x1.1-infected target cells in the assays. We also quantified the gD-1- and gB-1-specific antibody titers by ELISA in a subset of female mice (Table 1), as these are common targets of the human humoral immune response^16,17^. The Abs elicited in response to sublethal HSV-1 infection were primarily neutralizing (median neutralizing titer of 200 in both sexes) with little or no ADCC activity, and recognized both gD and gB.

### Vaccination and challenge of seropositive animals

**Fig. 2:**
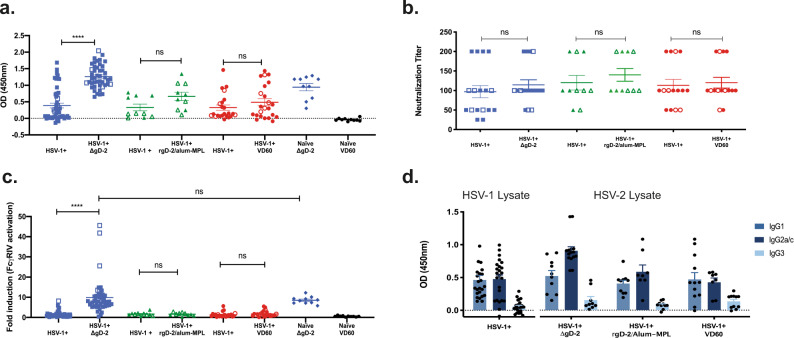
Vaccination of HSV-1-seropositive animals with ΔgD-2 boosts total HSV-2 and FcγRIV-activating antibodies. Beginning on day 21-post-HSV-1 challenge, female (closed symbols) and male (open symbols) mice were prime-boost vaccinated at 3-week intervals with 5 × 10^6^ pfu/mouse ΔgD-2; 5 µg of recombinant gD-2 with alum-MPL (rgD-2/alum-MPL) or an uninfected VD60 lysate. Additional controls included naïve age-matched mice vaccinated with ΔgD-2 or VD60 lysate. Serum was obtained on day 14 post-HSV-1 infection and one week following boost vaccination and assayed for **a** total HSV-2 IgG by ELISA (1:90,000 dilution); **b** neutralizing titer; and **c** fold-induction of FcγRIV activation (mFcγRIV ADCC Reporter Bioassay, Promega). **d** Total HSV-1-specific (left) and HSV-2-specific (right) IgG isotypes were assessed before (left) and after (right) boost vaccination. The asterisks (****) indicate *p* < 0.0001 by paired *t*-test comparing the post-vaccine versus post-HSV-1 antibody responses, ns indicates not significant. Data are shown as mean + SEM. The number of animals per group for Panels **a**–**c** were: HSV-1 seropositive (HSV-1^+^), ΔgD-2 *n* = 46; HSV-1^+^, rgD-2/alum-MPL *n* = 10; HSV-1^+^, VD60 *n* = 23^;^ Naïve, ΔgD-2 *n* = 20; Naïve, VD60 *n* = 20. For Panel **d**, the number of animals was 10 per vaccination group. Mice were from three independent experiments.

To evaluate the immunogenicity and efficacy of vaccines in this model, the HSV-1-seropositive mice were distributed into three different vaccination groups (ΔgD-2, rgD-2/alum-MPL, or control vaccine comprised of an uninfected VD60 cell lysate) based on ELISA titer as indicated in Fig. 1b. The seropositive mice were then prime-boost vaccinated intramuscularly at 3-week intervals with the first dose administered 21 days after HSV-1 infection. For comparison, HSV-1-unexposed (naïve) mice vaccinated either with ΔgD-2 or VD60 control lysate were included. The ability of the vaccines to boost total and functional antibody responses in HSV-1-seropositive mice was assessed by comparing serum obtained 1-week post-boost to serum obtained 14-days post-HSV-1 infection; HSV-2 (SD90)-infected cells were used as the targets to quantify HSV-2 IgG responses (ELISA), neutralizing titers and ADCC (murine FcγRIV activation) (Fig. 2). There were no differences in the pre-vaccination antibody responses comparing the mice assigned to each of the three vaccine groups, indicating comparable distribution (Fig. 2a–c). Vaccination with ΔgD-2 significantly boosted the total and FcγR-activating responses to levels similar to those observed in HSV-naïve mice vaccinated with ΔgD-2, but had no effect on the neutralizing titers (Fig. 2a–c). In contrast, there was no boosting of the total, neutralizing or ADCC responses in seropositive mice vaccinated with rgD-2/alum-MPL. Findings were similar and not statistically different in female and male mice (*p* > 0.05; closed and open symbols, respectively). Primary infection elicited IgG1 and IgG2 antibody responses and vaccination with ΔgD-2 boosted the IgG2 subclass response, which is the subclass primarily associated with FcγRIV activation and ADCC^18,19^ (Fig. 2d).

**Fig. 3:**
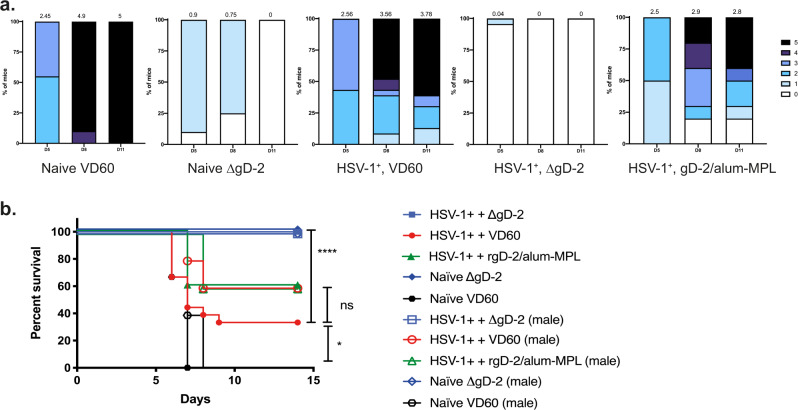
ΔgD-2-vaccinated HSV-1-seropositive animals are protected from subsequent HSV-2 skin challenge. Three weeks following boost vaccination, female (closed symbols) and male (open symbols) mice were challenged on the skin with 100 × LD90 (5 × 10^6^ pfu/mouse) of HSV-2 clinical isolate, SD90. **a** Disease scores (blinded) and **b** percentage survival following HSV-2 challenge. HSV-1^+^, ΔgD-2 *n* = 46; HSV-1^+^, rgD-2/alum-MPL *n* = 10; HSV-1^+^, VD60 *n* = 23; Naïve, ΔgD-2 *n* = 20; Naïve, VD60 *n* = 20 from three independent experiments. Survival was compared by Gehan–Breslow-Wilcoxon test (*p < 0.05, ****p < 0.0001, and ns not significant).

Pre-existing immunity to HSV-1 provided a small, but significant, survival benefit following challenge with 100-times the 90% lethal dose (100xLD90) of HSV-2 (SD90) compared to control-vaccinated naïve mice (*p* < 0.05), but all of these mice showed signs of disease, as illustrated by disease scores (Fig. 3a, b). Consistent with the lack of any boosting of the humoral immune response, there was no significant survival or disease score benefit when HSV-1-seropositive mice were vaccinated with rgD-2/alum-MPL. In contrast, ΔgD-2 provided complete protection from death following HSV challenge in both HSV-1-seropositive and naïve female and male mice against this high dose challenge and the mice showed little or no sign of disease (Figs 2 and 3).

### ΔgD-2 vaccination protects against HSV-2 spread to the sacral ganglia, but has no effect on the latent HSV-1 reservoir in the trigeminal ganglia

**Fig. 4:**
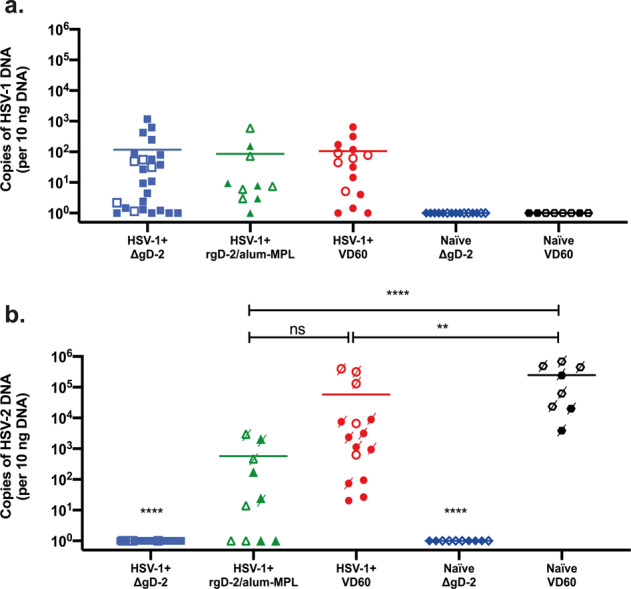
ΔgD-2 vaccination protects from establishing HSV-2 sacral ganglia latent reservoir but has no effect on the HSV-1 trigeminal ganglia reservoir. At the time of death or sacrifice (D14 post-HSV-2 challenge), trigeminal (**a**) and sacral (**b**) ganglia were isolated from female (closed symbols) and male (open symbols) mice, and HSV-1 and HSV-2 DNA quantified by qPCR using serotype-specific primers. Mice that succumbed to HSV-2 superinfection are indicated by a crossed-through symbol in **b**. HSV-1^+^, ΔgD-2 *n* = 25; HSV-1^+^, rgD-2-alum/MPL *n* = 10; HSV-1^+^, VD60 *n* = 15; Naïve, ΔgD-2 *n* = 10; Naïve, VD60 *n* = 8 from two independent experiments. The quantity of viral DNA detected was compared by ANOVA with correction for multiple comparison (**p < 0.01, ****p < 0.001, ns not significant), and means are indicated.

This superinfection model provided the opportunity to determine whether pre-existing HSV-1 immunity and/or vaccination modified the ability of HSV-2 to reach the sacral ganglia where latency is established and/or modulated the latent HSV-1 trigeminal ganglia reservoir. Using serotype-specific primers, comparable levels of HSV-1 DNA were detected in the trigeminal ganglia of HSV-1-seropositive, HSV-2-superinfected mice regardless of which of the vaccinations they received; no HSV-1 was detected in the trigeminal ganglia of the HSV-1-naïve, HSV-2-superinfected mice (Fig. 4a). No HSV-2 DNA was detected in sacral ganglia of HSV-1-seropositive or naïve mice vaccinated with ΔgD-2 (*p* < 0.0001 compared to all other groups). Less HSV-2 DNA was recovered from sacral ganglia of HSV-1-seropositive mice immunized with either rgD-2/alum-MPL or VD60 control lysate compared to naïve, control-vaccinated mice (*p* < 0.001 and *p* < 0.01, respectively). Among vaccinated seropositive animals, there was a trend towards less HSV-2 DNA in the HSV-1-seropositive mice that received rgD-2/alum-MPL compared to those that received the control vaccine, but this did not reach statistical significance.

### Vaccination of seropositive mice with dl5-29 boost ADCC, but not neutralizing titers

**Fig. 5:**
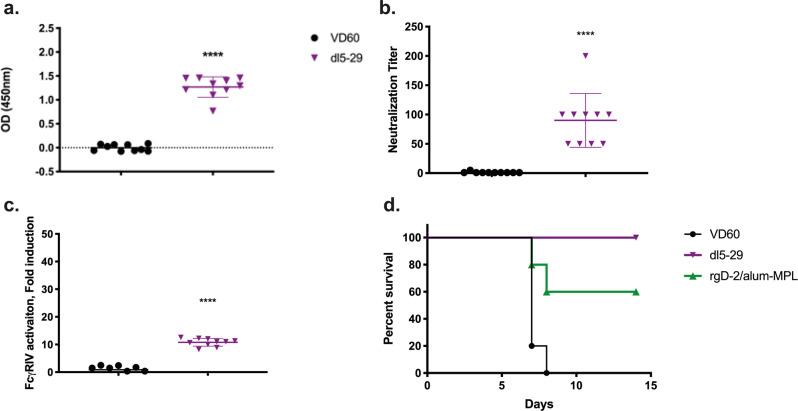
dl5-29 vaccination is immunogenic and protective in naïve animals challenged on the skin. Female C57BL6 mice were vaccinated with 5 × 10^6^ pfu/mouse of dl5-29 3 weeks apart. One week following boost vaccination, serum was collected and assessed for **a** total HSV-2-specific IgG by ELISA; **b** neutralizing titer; and **c** FcγRIV activation (mFcγRIV ADCC Reporter Bioassay, Promega). Three weeks following boost vaccination, mice vaccinated with dl5-29 or rgD-2/alum-MPL were challenged on the skin with a 1 × LD90 of HSV-2 SD90. Mice were monitored over 14 days for signs of disease and percentage survival is shown in **d**. Survival was compared by Gehan–Breslow–Wilcoxon test. The asterisks (****) indicate *p* < 0.0001, **p < 0.01, *p < 0.05 by Student’s *t*-test (**a**–**c**) or Gehan–Breslow–Wilcoxon test (**d**). Data are shown as mean + SEM. *n* = 10 for dl5-29 and VD60; *n* = 5 for rgD-2/alum-MPL.

**Fig. 6:**
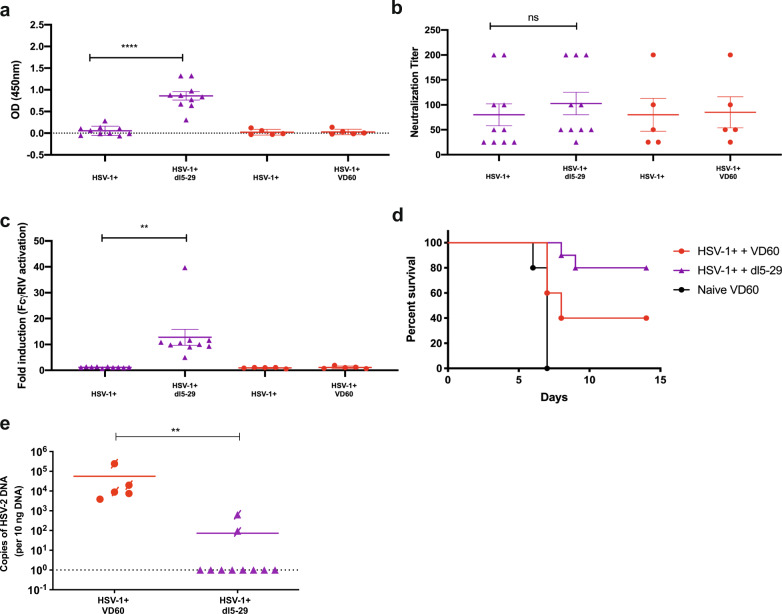
dl5-29 vaccination boosts total HSV-specific and ADCC but not neutralizing Ab. Female C57BL6 mice were infected intranasally with 5 × 10^4^ pfu/mouse of HSV-1 B^3^ × 1.1. Mice were monitored over 20 days for signs of disease and distress. At day 14 post-primary challenge, mice were bled retro-orbitally to obtain immune serum, which was tested for total HSV-1 specific IgG by ELISA and mice were assigned to vaccination groups based on HSV-1-specific IgG titers. Beginning on day 21-post-HSV-1 challenge, mice were prime-boost vaccinated at 3-week intervals with 5 × 10^6^ pfu/mouse dl5-29 or an uninfected VD60 lysate. Serum was obtained on day 14 post-HSV-1 infection and 1 week following boost vaccination and assayed for **a** total HSV-2 IgG by ELISA (1:90,000 dilution); **b** neutralizing titer; and **c** fold-induction of FcγRIV activation (mFcγRIV ADCC Reporter Bioassay, Promega). Three weeks following boost vaccination, mice were challenged on the skin with 100 × LD90 (5 × 10^6^ pfu/mouse) of HSV-2 clinical isolate, SD90. **d** Percentage survival following HSV-2 challenge. **e** At the time of death or sacrifice (D14 post-HSV-2 challenge), sacral ganglia were isolated and HSV-2 DNA was quantified by qPCR using serotype-specific primers. Mice that succumbed to HSV-2 superinfection are indicated by a crossed-through symbol. **a**–**c** Paired *t*-test comparing the post-vaccine versus post-HSV-1 antibody responses; **d** Gehan–Breslow-Wilcoxon test; **e** Fisher’s Exact Test. The asterisks (****) indicate *p* < 0.0001. **p < 0.01; ns indicates not significant. Data are shown as mean + SEM. *n* = 10 for dl5-29 and *n* = 5 for VD60.

To further evaluate this model of immunogenicity in HSV-1-seropositive mice, we evaluated a different candidate vaccine, the replication defective virus, *dl5-29*. Naïve female mice were prime-boost vaccinated with *dl5-29* (5 × 10^6^ pfu based on the titer on complementing cells, which is similar to the dose used in the guinea pig study)^10^. *Dl5-29* induced high titer HSV-specific Abs (Fig. 5a), comprised of both neutralizing (median HSV-2-neutralizing titer = 75) (Fig. 5b) and an ADCC response (median 10.3-fold, Fig. 5c), and resulted in complete protection from death following challenge with a 1 × LD90 of SD90 compared to 60% protection provided by rgD-2/alum-MPL (Fig. 5d). Studies were then conducted in HSV-1-seropositive mice. *Dl5-29* boosted the total, but not the neutralizing Ab response (Fig. 6a, b) and led to a significant increase in FcγRIV activation (Fig. 6c) in HSV-1-seropositive mice. This resulted in a nonsignificant increase in protection compared to HSV-1 seropositivity alone (80% vs 40%, *p* = 0.052, Fig. 6d) and a reduction in number of mice with detectable HSV-2 DNA in sacral ganglia (2/10 for HSV-1^+^, dl5-29-vaccinated versus 5/5 for HSV-1^+^, control-vaccinated, *p* = 0.007, Fisher’s exact test, Fig. 6e).

## Discussion

We established a model of HSV-2 superinfection of latently infected HSV-1-seropositive mice that reflects clinical experiences with natural infection and appears to better recapitulate the disappointing clinical trial outcomes with recombinant gD-2 vaccines compared to earlier guinea pig studies. Intranasal challenge of male or female mice with a sublethal dose of HSV-1 (B^3^ × 1.1) resulted in consistently high survival and seroconversion with variable HSV-1 IgG titers ranging from an endpoint dilution of 1000–270,000. Consistent with human studies, HSV-1 infection elicited Abs that targeted gD and gB and neutralized HSV-1 (median neutralization titer 200)^17,20^. The Abs cross-reacted with HSV-2 with a somewhat lower median neutralization titer of 100. The mice elicited only weak ADCC responses to natural infection, but the extent to which this parallels human response is not known, as most clinical studies have focused exclusively on binding and neutralizing responses. Variable ADCC responses were described in a small number of patients (pregnant women and their newborns) using a different assay and were associated with protection against viral dissemination in neonatal disease^21^.

Following challenge with a clinical isolate of HSV-2 at a relatively high dose (100 × LD90 in naïve mice), previous HSV-1 infection provided modest protection against severe disease (requiring euthanasia), but all of the mice exhibited signs of disease indicating no protection against infection. This parallels human experiences where studies have demonstrated that HSV-1 provides some protection against symptomatic HSV-2, but not against infection^22,23^. For example, data from the Chiron HSV Vaccine Study Group found that being seropositive for HSV-1 did not reduce the rate of HSV-2 infection, but increased the likelihood of asymptomatic compared to symptomatic seroconversion by a factor of 2.6 (*p* < 0.001)^22^. Similarly, analysis of a large nationally representative dataset (NHANES III) demonstrated that prior HSV-1 seropositivity reduced the likelihood of symptomatic HSV-2 disease, but did not protect against HSV-2 infection^24^.

In contrast to the preclinical studies in female guinea pigs, the murine model described here would have predicted the clinical trial outcomes with gD subunit vaccines, although the human trials used three vaccine doses, compared to the two used here^10,25^. Vaccination of HSV-1-seropositive female guinea pigs (infected intranasally with the laboratory-adapted viral strain, KOS) with recombinant, adjuvanted gD-2 significantly boosted the neutralizing antibody titers against both HSV-1 and HSV-2, and protected HSV-1-seropositive guinea pigs from acute and recurrent disease after intravaginal challenge with HSV-2 (333), a laboratory-adapted strain^10^. These findings, however, were not predictive of the clinical trial results with gD-AS04 in seropositive individuals. A serodiscordant couples study that included HSV-1-seropositive individuals at risk for HSV-2 found that there was no boosting of neutralizing antibody titers and the vaccine did not protect the HSV-1-seropositive men or women. Notably, there was also no protection in seronegative men, which were not included in the guinea pig studies, but ~70% protection in doubly-seronegative women^8^. In a subsequent field study, which enrolled 8323 doubly-seronegative women, the overall vaccine efficacy against genital disease was only 20% (95% confidence interval [CI], −29 to 50)^9^. These disappointing clinical trial outcomes with gD subunit vaccines despite the efficacy observed in the established preclinical female intravaginal small animal models challenge the predictive value of these traditional models, and raise questions about the reliance on neutralizing Abs as a correlate of vaccine efficacy.

The murine superinfection model described here more closely recapitulates what was observed in the clinical trial, as evidenced by the failure of the subunit gD-2 vaccine to boost total or neutralizing antibody titers in HSV-1-seropositive mice or to protect mice against HSV-2 superinfection^8^. The two models differ in several aspects including species (mouse versus guinea pig), challenge site (flank skin versus vaginal), and challenge with clinical rather than laboratory-adapted isolates. Which of these differences contributed to the greater recapitulation of the clinical trial outcomes will require further study. Skin challenge is reflective of human HSV-2 disease, which more commonly presents on external genital skin rather than intravaginally or within the cervix^26–28^, and allows for evaluation of both males and females. Isolates vary in their ability to cause disease in animal models and this may reflect, in part, how extensively they have been passaged in the laboratory^29^. Sequence differences between strains may also contribute to virulence, although sequencing data suggest relatively high conservation among HSV-2 strains. For example, in a recent study, 34 low-passage clinical and laboratory-adapted HSV-2 isolates were sequenced and the maximum nucleotide divergence between strains was only 0.4%. The sequences closely resembled SD90, which was used as the challenge strain in these studies^30^.

In contrast to results obtained with rgD-2/alum-MPL, the novel ΔgD-2 vaccine, which elicits a predominantly non-neutralizing, FcγRIV-activating ADCC immune response, was fully protective in both seronegative and HSV-1-seropositive female and male mice. Pre-existing HSV-1 infection did not interfere with the ability of the ΔgD-2 vaccine to elicit IgG2 subclass-switched Abs; the subclass associated with ADCC^18^. The ADCC responses elicited in the HSV-1-seropositive mice were similar to those elicited in response to the vaccine in naïve mice, and the complete protection against death caused by HSV and the establishment of latency was identical to what was previously observed in female (vaginal or skin challenge) and male (skin challenge) naïve mice^12–14^. The findings further support the notion that ADCC may provide a better correlate of immune protection than neutralizing Abs, which were elicited in response to primary HSV-1 infection as well as to the rgD-2/alum-MPL vaccine, but provided only partial cross-serotype protection against subsequent HSV-2 challenge and latency. The importance of ADCC is further highlighted by results obtained with *dl5-29*. DL5-29 provides 100% protection from death in naïve mice following lethal challenge with HSV-2, compared to the 60% protection afforded by gD-2/alum-MPL. This indicates that both vaccines provide protection against an LD90 challenge with HSV-2, although studies in HSV-1-seropositive animals used a 100 × LD90. *Dl5-29* vaccination of HSV-1-seropositive mice boosted the ADCC, but not the neutralizing response, which was associated with greater protection against HSV-2 superinfection compared to HSV-1-seropositivity alone. The absence of any increase in neutralizing titers is consistent with the Phase 1 *dl5-29* study where no sustained boost in neutralizing titers was observed^15^.

The ΔgD-2 vaccine completely prevented the establishment of latency in the sacral ganglia following HSV-2 challenge in both seronegative and seropositive mice, but had no discernible effect on the size of the HSV-1 latent reservoir in the trigeminal ganglia. This presumably reflects the fact that latent virus does not express viral proteins that are targets of the ADCC response and suggests that vaccination will not kill latently infected neurons. A limitation of the murine model, however, is that it does not allow for the assessment of the effects of the vaccine on viral reactivation or recurrent disease, as spontaneous reactivation has not been observed in murine studies. This contrasts with the guinea pig, where spontaneous recurrences do occur thus providing a potentially important model to address this question. However, to date, the model has not proven predictive of clinical trial results.

In conclusion, we have established and validated a murine model of HSV-1 seropositivity that provides insight in to the primary antibody response to HSV infection, as well as a tractable model to study the efficacy of vaccines in the context of pre-existing anti-HSV antibody responses. The findings support further development of ΔgD-2 as a promising candidate for vaccination of both HSV-1-seropositive and -seronegative individuals, which, in contrast to the rgD-2/alum-MPL vaccine, boosted total HSV-specific Abs and generated ADCC responses that completely protected the mice from a high dose lethal HSV-2 challenge. The findings suggest that this murine superinfection model should be incorporated into the preclinical evaluation of future HSV-vaccine candidates.

## Methods

### Mice and ethics statement

Age-matched female and male C57BL/6 mice were purchased from the Jackson Laboratory (JAX, Bar Harbor, ME). The use of animals was approved by the Institutional Animal Care and Use Committee at the Albert Einstein College of Medicine, Protocol 2018-0504, and all relevant ethical regulations were complied with.

### Cell lines and viruses

Vero (Green Monkey Kidney cells line, ATCC) and VD60^31^ cells were grown in DMEM (Invitrogen, Carlsbad, CA) supplemented with 10% FBS (Hyclone, Logan, UT) and 1% penicillin-streptomycin (Invitrogen). The HSV-2 ΔgD-2 vaccine strain was propagated and titered in VD60 cells, which express HSV-1 gD under its native promoter, thus yielding phenotypically complemented, but genotypically gD-null progeny. The *dl5-29* candidate vaccine propagated and titered on V529 complementing cells^32,33^. The HSV-1 clinical isolate, B^3^ × 1.1, was obtained from the Montefiore Clinical Virology Lab^13,14,33^, HSV-2(G) from Bernard Roizman (U. of Chicago)^34^, and SD90^33^. These isolates were propagated and titered on Vero cells.

### HSV infections and immunizations

Mice (4–6 weeks of age) were anesthetized with isoflurane and infected intranasally with 5 × 10^4^ pfu/mouse of HSV-1 B^3^ × 1.1 in 10 µL PBS (Day 0). Mice were monitored daily for signs of disease, and were sacrificed if they showed signs of distress or neurological disease (hunching, decreased activity, not eating or consistent leaning or circling). Blood was obtained retro-orbitally on Day 14 to quantify HSV Abs by ELISA (described below). Based on the convalescent antibody levels, mice were assigned to one of three subsequent vaccination groups such that the titers across groups were comparable, and then mice were vaccinated on Day 21 (prime) and Day 42 (boost) intramuscularly (i.m.) with 5 × 10^6^ pfu of ΔgD-2 (based on VD60 cell titer), 5 µg of recombinant gD-2 protein (Molecular Therapeutics Core Facility, Albert Einstein College of Medicine) generated in mammalian cells as described in ref. ^15^ combined with 150 µg alum (Imject Alum, Pierce Biotechnology, Rockland, IL) and 12.5 µg MPL (Invivogen, San Diego, CA) (rgD-2/alum-MPL) or an uninfected VD60 cell lysate as a control. In a subsequent study, HSV-1-seropositive mice were assigned to groups that received either 5 × 10^6^ pfu of *dl5-29* or a VD60 control vaccine. Blood was obtained on Day 49 for post-boost immune assays and on Day 63, mice were challenged with 100 × LD90 dose (5 × 10^6^ pfu/mouse) of HSV-2 SD90 on depilated, gently abraded flank skin^12–14^. Briefly, following hair removal, skin was scratched with an emery board for ~20 strokes. This treatment did not break the skin. Virus was applied (5 µL) to abraded skin and mice were monitored daily for signs of epithelial and/or neurological disease and scored (blinded) as follows: (1) Erythema at inoculation site; (2) Spread to distant site, zosteriform lesions, edema; (3) Ulceration, epidermal spread, hind limb weakness, or paresis; (4) Hind limb paralysis; and (5) Death. Mice were euthanized at a score of 4 and assigned a score of 5 the following day.

### Immunologic assays

To assess total HSV-1 and 2 IgG levels, ELISA plates were coated with lysates of Vero cells infected with HSV-1 B^3^ × 1.1 or HSV-2(G) at an MOI of 0.1 for 24 h or uninfected Vero cell lysates as control. Serial 3-fold dilutions of serum (starting with 1:1000) were incubated with coated plates overnight at 4 °C, and bound IgG was quantified using a biotin-labeled secondary antibody (BD Pharmingen, CA). HSV-1 and 2 isotypes were assessed in the same way, using isotype-specific (IgG1, IgG2a/b, and IgG3) biotin-labeled secondary Abs. To quantify gD- or gB-specific antibody responses, ELISA assays were also performed using recombinant proteins. ELISA plates were coated with 10 ng/well of gB-1 or gD-1 protein (Molecular Therapeutics Core Facility, Albert Einstein College of Medicine) and incubated with serum as described above^14^. Neutralizing titers were determined by plaque reduction assay. Serial 2-fold dilutions of heat-inactivated serum were incubated with virus (50 pfu/well) for 1 h at 37 °C and then the mixture was added to a Vero cell monolayer for 1 h at 37 °C. Cells were fixed with methanol and stained with Giemsa after a 48-h incubation. Plaques were counted and the neutralization titer was defined as the highest dilution to result in a 50% reduction in plaque numbers. FcγRIV activation was determined using the mFcγRIV ADCC Reporter Bioassay (Promega, Madison, WI). Vero cells were infected with HSV-1 B^3^ × 1.1 or HSV-2 SD90 at an MOI of 0.1 for 12 h as targets for the assay, transferred to white, flat-bottomed 96-well plates and incubated with heat-inactivated serum from immunized mice (1:5 dilution in DMEM) for 15 min at room temperature. FcγRIV reporter cells were added for 6 h at 37 °C 5% CO_2_. FcγRIV activation was detected by the addition of luciferin substrate. Plates were read in a SpectraMax M5^e^ (Molecular Devices). Fold-induction was calculated relative to luciferase activity in the absence of serum.

### Quantification of viral DNA in neuronal tissue by quantitative PCR

At the time of euthanasia (when mice succumbed to disease or day 14 post-HSV-2 challenge); trigeminal and sacral nerve tissue was extracted and DNA was isolated using the Qiagen Blood and Tissue DNA isolation kit (Qiagen). Ten nanogram of DNA per sample was loaded and primers and probes specific for HSV-1 and HSV-2 gB were used to quantify HSV DNA (HSV-1 forward primer sequence 5′- GCAGTTTACGTACAACCACATACAGC -3′, HSV-2 forward primer sequence 5’ - TGCAGTTTACGTATAACCACATACAGC - 3’; HSV-1 and 2 reverse primer sequence 5′- AGCTTGCGGGCCTCGTT -3′, HSV-1 probe sequence 5′- CGGCCCAACATATCGTTGACATGGC -3′, HSV-2 probe sequence 5’- CGCCCCAGCATGTCGTTCACGT -3’) (Supplementary Table 1)^35^. Mouse β actin was used as a loading control (Applied Biosystems, Foster City, CA), and qPCR was run in an Applied Biosystems QuantStudio 7 Flex. Based on a standard curve, this assay consistently detected copy numbers greater than or equal to 4. Samples with fewer than four copies detected were considered negative^12–14^.

### Statistical analysis

Analyses were performed using GraphPad Prism version 8.2.1 software (GraphPad Software Inc. San Diego, CA). A *p*-value of 0.05 was considered statistically significant. Survival curves were compared using the Gehan–Breslow–Wilcoxon test; other results were compared using Student’s paired and unpaired *t*-tests or ANOVA as indicated.

### Reporting summary

Further information on experimental design is available in the Nature Research Reporting Summary linked to this article.

## Supplementary information

Supplementary Table

Reporting Summary

## Data Availability

The authors declare that all data supporting the findings of this study are available within the manuscript.

## References

[1] Roberts CM, Pfister JR, Spear SJ (2003). Increasing proportion of herpes simplex virus type 1 as a cause of genital herpes infection in college students. Sex. Transm. Dis..

[2] Looker KJ (2015). Global estimates of prevalent and incident herpes simplex virus type 2 infections in 2012. PLoS ONE.

[3] Lafferty WE, Downey L, Celum C, Wald A (2000). Herpes simplex virus type 1 as a cause of genital herpes: impact on surveillance and prevention. J. Infect. Dis..

[4] Blower S, Ma L (2004). Calculating the contribution of herpes simplex virus type 2 epidemics to increasing HIV incidence: treatment implications. Clin. Infect. Dis..

[5] Freeman EE (2007). Proportion of new HIV infections attributable to herpes simplex 2 increases over time: simulations of the changing role of sexually transmitted infections in sub-Saharan African HIV epidemics. Sex. Transm. Infect..

[6] Looker KJ (2015). Global and regional estimates of prevalent and incident herpes simplex virus type 1 infections in 2012. PLoS ONE.

[7] Looker, K. J., Elmes, J., Gottlieb, S. L. & Schiffer, J. T. Effect of HSV-2 infection on subsequent HIV acquisition: an updated systematic review and meta-analysis, *Lancet Infect.*10.1016/S1473-3099(17)30405-X (2017). 10.1016/S1473-3099(17)30405-X PMC5700807 28843576

[8] Stanberry LR (2002). GlaxoSmithKline Herpes Vaccine Efficacy Study Group, Glycoprotein-D-adjuvant vaccine to prevent genital herpes. N. Engl. J. Med..

[9] Belshe RB (2012). Herpevac trial for women, efficacy results of a trial of a herpes simplex vaccine. N. Engl. J. Med..

[10] Hoshino Y (2009). Protection from herpes simplex virus (HSV)-2 infection with replication-defective HSV-2 or glycoprotein D2 vaccines in HSV-1-seropositive and HSV-1-seronegative guinea pigs. J. Infect. Dis..

[11] Klein RJ, Friedman-Kien AE, Brady E (1978). Latent herpes simplex virus in ganglia of mice after primary infection and reinoculation at a distant site. Arch. Virol..

[12] Petro, C., González, P. A., Cheshenko, N. & Jandl, T. Herpes simplex type 2 virus deleted in glycoprotein D protects against vaginal, skin and neural disease, *eLife*10.7554/eLife.06054.001 (2015). 10.7554/eLife.06054 PMC4352706 25756612

[13] Petro CD (2016). HSV-2 ΔgD elicits FcγR-effector antibodies that protect against clinical isolates. JCI Insight.

[14] Burn, C. et al. An HSV-2 single-cycle candidate vaccine deleted in glycoprotein D, ΔgD-2, protects male mice from lethal skin challenge with clinical isolates of HSV-1 and HSV-2. *J. Infect. Dis.* **217**, 754–758 (2018). 10.1093/infdis/jix628 PMC5853290 29216362

[15] Dropulic LK (2019). A randomized, double-blinded, placebo-controlled, phase 1 study of a replication-defective herpes simplex virus (HSV) type 2 vaccine, HSV529, in adults with or without HSV infection. J. Infect. Dis..

[16] Cairns TM (2014). Dissection of the antibody response against herpes simplex virus glycoproteins in naturally infected humans. J. Virol..

[17] Cairns TM (2015). Patient-specific neutralizing antibody responses to herpes simplex virus are attributed to epitopes on gD, gB, or both and can be type specific. J. Virol..

[18] Nimmerjahn F, Bruhns P, Horiuchi K, Ravetch JV (2005). FcgammaRIV: a novel FcR with distinct IgG subclass specificity. Immunity.

[19] Huber VC (2006). Distinct contributions of vaccine-induced immunoglobulin G1 (IgG1) and IgG2a antibodies to protective immunity against influenza. Clin. Vaccin. Immunol..

[20] Criscuolo E (2019). Cell-to-cell spread blocking activity is extremely limited in the sera of herpes simplex virus 1 (HSV-1)- and HSV-2-infected subjects. J. Virol..

[21] Kohl S (1989). Neonatal antibody-dependent cellular cytotoxic antibody levels are associated with the clinical presentation of neonatal herpes simplex virus infection. J. Infect. Dis..

[22] Langenberg AG, Corey L, Ashley RL, Leong WP, Straus SE (1999). A prospective study of new infections with herpes simplex virus type 1 and type 2. Chiron HSV Vaccine Study Group. N. Engl. J. Med..

[23] Looker KJ (2005). A systematic review of the epidemiology and interaction of herpes simplex virus types 1 and 2. Sex. Transm. Infect..

[24] Xu F (2002). Seroprevalence and coinfection with herpes simplex virus type 1 and type 2 in the United States, 1988-1994. J. Infect. Dis..

[25] Awasthi S (2014). Protection provided by a Herpes Simplex Virus 2 (HSV-2) glycoprotein C and D subunit antigen vaccine against genital HSV-2 infection in HSV-1-seropositive guinea pigs. J. Virol..

[26] Gupta R, Warren T, Wald A (2007). Genital herpes. Lancet.

[27] Tanton C (2011). Patterns of herpes simplex virus shedding over 1 month and the impact of acyclovir and HIV in HSV-2-seropositive women in Tanzania. Sex. Transm. Infect..

[28] Keller MJ (2012). Changes in the soluble mucosal immune environment during genital herpes outbreaks. J. Acquir. Immune Defic. Syndr..

[29] Kollias CM, Huneke RB, Wigdahl B, Jennings SR (2014). Animal models of herpes simplex virus immunity and pathogenesis. J. Neurovirol..

[30] Newman RM (2015). Genome sequencing and analysis of geographically diverse clinical isolates of herpes simplex virus 2. J. Virol..

[31] Ligas MW, Johnson DC (1988). A herpes simplex virus mutant in which glycoprotein D sequences are replaced by beta-galactosidase sequences binds to but is unable to penetrate into cells. J. Virol..

[32] Da Costa XJEA, Kramer MF, Zhu J, Brockman MA, Knipe DM (2000). Construction, phenotypic analysis, and immunogenicity of a UL5/UL29 double deletion mutant of herpes simplex virus 2. J. Virol..

[33] Dudek TE, Torres-Lopez E, Crumpacker C, Knipe DM (2011). Evidence for differences in immunologic and pathogenesis properties of herpes simplex virus 2 strains from the United States and South Africa. J. Infect. Dis..

[34] Ejercito PM, Kieff ED, Roizman B (1968). Characterization of herpes simplex virus strains differing in their effects on social behaviour of infected cells. J. Gen. Virol..

[35] Namvar L, Olofsson S, Bergström T, Lindh M (2005). Detection and typing of Herpes Simplex virus (HSV) in mucocutaneous samples by TaqMan PCR targeting a gB segment homologous for HSV types 1 and 2. J. Clin. Microbiol..

