# Supplementary material for: Model of vaccine efficacy against HSV-2 superinfection of HSV-1 seropositive mice demonstrates protection by antibodies mediating cellular cytotoxicity
Source: NPJ Vaccines. 2020 May 7;5:35. doi: 10.1038/s41541-020-0184-7 (PMC7206093; doi:10.1038/s41541-020-0184-7)
Supplement: Supplementary file 1 — Supplementary Table [file 41541_2020_184_MOESM1_ESM.pdf]

Name	Sequence (5’-3’)
HSV-1 gB forward primer	GCAGTTTACGTACAACCACATACAGC
HSV-2 gB forward primer	TGCAGTTTACGTATAACCACATACAGC
HSV-1 and HSV-2 gB reverse primer	AGCTTGCGGGCCTCGTT
HSV-1 gB probe	CGGCCCAACATATCGTTGACATGGC
HSV-2 gB probe	CGCCCCAGCATGTCGTTCACGT

Supplementary Table 1. HSV-specific primer and probe sequences for qPCR.
